# Application of Electroencephalography Sensors and Artificial Intelligence in Automated Language Teaching

**DOI:** 10.3390/s24216969

**Published:** 2024-10-30

**Authors:** Yanlin Chen, Wuxiong Wang, Shen Yan, Yiming Wang, Xinran Zheng, Chunli Lv

**Affiliations:** China Agricultural University, Beijing 100083, China; 2022314040520@cau.edu.cn (Y.C.); wangwuxiong@cau.edu.cn (W.W.); yanshen2022@cau.edu.cn (S.Y.); yimingwang19@cau.edu.cn (Y.W.); zhengxinran@cau.edu.cn (X.Z.)

**Keywords:** EEG sensors in education, real-time cognitive monitoring, sensor-based learning assessment, differential adaptive learning models, deep learning

## Abstract

This study developed an automated language learning teaching assessment system based on electroencephalography (EEG) and differential language large models (LLMs), aimed at enhancing language instruction effectiveness by monitoring learners’ cognitive states in real time and personalizing teaching content accordingly. Through detailed experimental design, the paper validated the system’s application in various teaching tasks. The results indicate that the system exhibited high precision, recall, and accuracy in teaching effectiveness tests. Specifically, the method integrating differential LLMs with the EEG fusion module achieved a precision of 0.96, recall of 0.95, accuracy of 0.96, and an F1-score of 0.95, outperforming other automated teaching models. Additionally, ablation experiments further confirmed the critical role of the EEG fusion module in enhancing teaching quality and accuracy, providing valuable data support and theoretical basis for future improvements in teaching methods and system design.

## 1. Introduction

In the context of contemporary globalization, language learning is recognized as an interdisciplinary process within a multilingual, multicultural reality [1,2]. As a fundamental discipline, the innovation of teaching and assessment methods plays a critical role in enhancing the quality of education [3,4]. Effective evaluation of learning outcomes is considered a crucial component of the teaching process, essential for improving student learning outcomes and optimizing teaching practices [5]. Multidimensional assessments are necessary, requiring a variety of tools and technologies [6]. The use of advanced technologies to assess learning outcomes has become a focal point of research [7], especially in language learning. This paper proposes an automated language learning teaching assessment system based on electroencephalography (EEG) and differential language large models (LLMs), aiming to explore new pathways combining brain signal analysis with big data processing techniques to optimize the teaching and assessment processes of language learning.

Traditionally, language learning has been viewed as a “black box”, focusing only on external inputs (such as students, teachers, resources) and expected outputs (knowledge and skills) [8], with the responsibility for assessment lying entirely with teachers. The primary assessment methods have relied on paper-based tests and subjective evaluations by teachers [9], which, while effective, come with numerous limitations. For instance, paper-based tests typically assess students’ language levels at specific time points, failing to comprehensively reflect the ongoing language learning process or real-time progress [10]. Moreover, traditional assessment methods are operationally cumbersome and time-consuming, potentially impacting teaching efficiency and learner motivation. With the evolution of educational technology, there is a pressing need to develop more efficient, objective tools for language learning assessment.

EEG technology, as a method capable of real-time monitoring of brain activity, has been extensively applied in medical and psychological research [11]. The integration of EEG into the educational field, particularly for assessing learning outcomes, provides a novel research tool [12,13]. Bashir et al. [14] suggest that compared to traditional assessment methods, EEG technology is less susceptible to assessor bias and can produce quantitative data. By analyzing changes in brainwaves during the learning process, EEG can more accurately reflect learners’ cognitive states and efficiency, yet it faces challenges such as significant computational errors. Ramires et al. [15] developed a machine learning tool using a Multivariate Linear Regression (MLR) model to predict the cognitive performance of university students under different learning modalities. This study involved collecting EEG signals using the OpenBCI (OpenBCI is an open-source brain–computer interface platform), system, cleaning data with the automatic speech recognition algorithm, calculating various power ratios, selecting significant features through correlation analysis, and inputting these features into the MLR model, achieving a maximum accuracy of 85.67%. Al-Nafjan et al. [16] introduced an EEG-based brain–computer interface (BCI) system to monitor student attention during online learning. Features were extracted using Fast Fourier Transform, achieving 96% accuracy with the Random Forest (RF) algorithm. Fuentes et al. [17] presented an EEG platform for real-time monitoring of student attention, showing that the power spectral density (PSD) of the Beta band significantly correlates with academic performance (r = 0.53, *p* = 0.003), outperforming traditional subjective assessments by teachers. By analyzing changes in brainwaves during the learning process, a more precise understanding of learners’ cognitive states and efficiency can be achieved, thus providing real-time feedback and personalized guidance for teaching.

Simultaneously, LLMs, such as Generative Pre-Trained (GPT) and Bidirectional Encoder Representations from Transformers (BERT), have demonstrated remarkable capabilities in language understanding and generation tasks [18]. These models, trained on extensive datasets, are able to capture the deep structures of language, providing powerful tools for language teaching. Abedi et al. [19] applied LLMs and chatbots in courses, validating their effectiveness in answering complex questions, autonomous learning, and providing immediate feedback. They also extended the functionality of chatbots through integration with smart prompts and plugins like Wolfram Alpha, enhancing teaching quality. Li et al. [20] examined the capabilities of LLMs in mathematics, writing, programming, reasoning, and knowledge questioning, noting that while traditional education relies on teachers, online education, although cost-effective, offers limited personalized learning. LLMs, like ChatGPT, excel in understanding, reasoning, and problem-solving, bringing new opportunities to the education sector. Bonner et al. [21] noted that LLMs can provide creative activities in language classes, understanding dialogues, and generating texts, offering personalized teaching materials and feedback for language learning. However, despite the outstanding performance of these models in language tasks, integrating them effectively to assess and enhance language learning remains a challenge. The main contributions of this paper include the following:

1. EEG fusion module: The core of this module lies in using EEG data to monitor changes in brain activity during the language learning process. This monitoring can provide crucial information about learners’ cognitive load, attention distribution, and emotional states, essential for assessing learning outcomes.
2. Differential LLMs: Traditional language models generally apply the same processing and response mechanisms to all users, whereas differential language large models adjust according to each learner’s specific circumstances (such as learning history, cognitive abilities, and personal preferences). Differential LLMs, by analyzing the interaction data between learners and the model, can learn and simulate the most effective teaching strategies to accommodate unique learner needs. For example, the model might employ more intuitive and repetitive teaching methods for beginners, while adopting more complex and challenging tasks for advanced learners.
3. Differential loss function: During the model training process, the differential loss function optimizes the learning path of the model, ensuring that it not only learns general linguistic rules but also captures specific patterns related to individual learners.

In summary, through technological innovation, this system achieves a deep understanding and real-time, dynamic assessment of the language learning process, providing a novel solution for the field of language education.

## 2. Related Work

### 2.1. EEG in Education

The introduction of EEG technology has provided a new perspective for monitoring and assessing the learning process amid the rapid development of educational technologies [22,23,24]. As a non-invasive technique capable of real-time monitoring of brain activity, EEG captures the electrical activity on the scalp, reflecting the functional state of the brain [25]. In the field of education, the application of EEG allows researchers to directly observe changes in brain activity during learning activities [16,26], closely linked to cognitive functions and emotional states, providing valuable physiological data for a deep understanding of the learning process.

EEG signals are primarily obtained by measuring the voltage differences from the electrical activity of brain neurons [27]. By placing multiple electrodes on the scalp, brainwaves across different frequency ranges are captured, reflecting various cognitive states [28,29]. The basic processing of EEG signals includes amplification, filtering, digitization, and feature extraction and classification through various algorithms. Brainwaves are categorized into several basic types based on frequency: δ (Delta, 1–4 Hz), θ (Theta, 4–8 Hz), α (Alpha, 8–13 Hz), β (Beta, 13–30 Hz), and γ (Gamma, above 30 Hz). Changes in α and θ waves are commonly used to assess learners’ attention levels and cognitive load.

A key aspect of EEG signal analysis is understanding the relationship between these waveforms and learning behaviors. For example, the amplitude of α waves is usually associated with relaxed and quiet wakeful states, while a reduction in α waves during cognitive tasks often indicates focused attention. Mathematically, this change can be quantified by calculating the power spectral density of α waves in different states, represented by the following equation:

(1) $$PSD\left(f\right)=\lim_{T\to \infty }\frac{1}{T}{\left|\int_{-T/2}^{T/2}x\left(t\right)e^{-i2\pi ft}dt\right|}^{2},$$

where x(t) is the EEG signal in the time domain, and *f* is the frequency, with PSD representing the power spectral density.

In this study, particular attention is paid to how EEG technology combined with differential LLMs can optimize language learning. The real-time monitoring capability of EEG enables the capture of subtle changes in brain activity during language teaching [30], reflecting the learner’s cognitive state and learning efficiency. By correlating EEG data with performance in language learning tasks (such as language comprehension and application ability tests generated through LLMs), a more accurate assessment and prediction of learning outcomes can be achieved. Furthermore, the application of EEG in personalized teaching also demonstrates tremendous potential [31]. By analyzing the brainwave activity of learners during language learning tasks, personalized learning plans can be customized. For instance, a reduction in α wave activity during a task may indicate excessive cognitive load. Based on such information, educators can timely adjust the difficulty or method of teaching to reduce cognitive load, thereby enhancing learning efficiency.

### 2.2. Large Language Models in Language Teaching

LLMs, such as GPT-3 [32] and BERT [33], have become significant representatives of natural language processing (NLP) technologies [34]. These models’ core capabilities lie in understanding and generating natural language, achieved through training on large-scale datasets to learn the deep structure and semantics of language. In the realm of language teaching, the application of LLMs has paved new pathways for instruction and learning, particularly in providing dynamic interactive environments and personalized learning support [21,35,36].

LLMs are typically based on the Transformer architecture [37], which is characterized by the self-attention mechanism. This mechanism allows the model to assign different attention weights to different parts of the input data, thereby enhancing text comprehension and generation. The calculation formula for self-attention is expressed as

(2) $$\mathrm{Attention}\left(Q,K,V\right)=\mathrm{softmax}\left(\frac{QK^{T}}{\sqrt{d_{k}}}\right)V,$$

where *Q*, *K*, and *V* represent the query, key, and value matrices, respectively, and $d_{k}$ is the dimension of the key vector. This mechanism enables LLMs to excel in handling long-distance dependencies and complex sentence structures. Within the context of this research, the integration of EEG and differential language large models into an automated system for assessing language learning significantly optimizes teaching outcomes and assessment accuracy. The real-time monitoring of learners’ brain activity, correlated with the outputs from LLMs, facilitates more precise evaluations of cognitive states and language learning progress.

LLMs provide learners with an interactive mode of language learning by generating text that is fluent and closely resembles natural language [38]. This not only allows learners to practice language skills in an environment akin to real-life dialogues but also enables immediate error correction and deeper understanding through model feedback. The model’s generative capability facilitates the construction of complex dialogue scenarios, enhancing learners’ adaptability and application skills in varied social and professional settings [39]. The basic model for generating dialogue is represented as

(3) GeneratedText=LM(contextinputs).

Here, LM denotes the language model, and context inputs are customized based on the learner’s historical and real-time EEG data. Differential language large models adapt the difficulty and type of learning content based on the specific needs of learners, offering a truly personalized learning experience [40]. This personalization is achieved by analyzing the learner’s progress, preferences, and cognitive load detected through EEG monitoring. The model dynamically adjusts the content to ensure that each learner studies at the most suitable pace and difficulty, summarized by the following formula:

(4) CustomizedContent=f(EEGdata,LearningHistory).

Here, *f* is a mapping function that takes EEG data and the learner’s historical learning context as inputs and outputs the most suitable teaching content for the learner’s current cognitive state and learning needs. By analyzing the interactions between learners and LLMs, real-time evaluations of various aspects of language proficiency [41], such as grammatical accuracy, vocabulary richness, and fluency, are conducted [42,43]. These evaluations provide educators with detailed feedback on learners’ progress, supporting further instructional decisions [44]. The evaluation process is automated to analyze the accuracy and richness of learners’ responses, with the assessment formula presented as

(5) LanguageProficiency=Evaluate(learner’sresponses,model’sfeedback).

Through this method, combining EEG technology with the capabilities of LLMs not only enhances the efficiency and quality of language learning but also significantly increases the personalization and adaptability of teaching through customized learning and assessment methods.

## 3. Materials and Method

### 3.1. Data Collection and Preprocessing

#### 3.1.1. Test Data Collection

**Participant Consistency.** This study recruited 200 participants aged between 18 and 24 years, divided into five groups, as shown in Table 1. All participants underwent auditory and cognitive ability tests to ensure they met the basic requirements for participation, thereby ensuring the validity and reliability of the experimental data. Regarding the participants’ sociodemographic and ethnic characteristics, we collected multidimensional information, including gender and educational background. All participants were university students, covering various academic disciplines, including humanities, social sciences, and engineering. This diversity enabled us to evaluate language learning differences across different academic backgrounds. For the language studied, all participants were learning the same new language—Spanish was chosen for this experiment. This setting ensured that all participants started at the same baseline, making the assessment of learning outcomes more fair and scientifically rigorous.

**Collection Description.** Multiple repetitions of the experiment were conducted to ensure robustness and reliability of the results. All experiments were conducted in a laboratory environment to control for external interference and ensure data accuracy. Each learning session lasted 10–15 min, with an overall data collection period of six months (January to July 2024) to ensure an adequate sample size and statistical representativeness. All experimental data will be kept strictly confidential, and the experiment followed the ethical guidelines provided by the ethics committee to ensure participants’ privacy.

The following five Spanish learning themes were designed:

1. Deportes (Sports): Learning vocabulary related to different sports, such as soccer, basketball, tennis, etc., covering rules, equipment, and common conversations and expressions used in sports contexts.
2. Viajes y turismo (Travel and Tourism): Learning vocabulary and expressions related to travel, including how to ask for directions, book hotels, buy tickets, and apply language in various travel scenarios.
3. Restaurantes y comida (Restaurants and Food): Learning common expressions used when ordering food, asking about the menu, and communicating with servers, while also familiarizing students with vocabulary related to different cuisines, ingredients, and food culture.
4. Compras y mercado (Shopping and Market): Covering language skills used in shopping scenarios, such as asking for prices, comparing products, discussing discounts, payment methods, and common items and expressions found in markets.
5. Salud y bienestar (Health and Wellness): Learning medical and health-related expressions used in hospitals, pharmacies, etc., including how to describe symptoms, ask about medications, and engage in common conversations in medical services.

Although these themes vary in context, the language requirements are consistent, as they all require students to master foundational vocabulary, common expressions, and grammar rules specific to each scenario. These themes aim to prepare students to use language in various real-life situations, equipping them with the necessary linguistic tools to handle diverse communication needs. After completing each theme, students will be tested on 10 different aspects, such as vocabulary tests, pronunciation practice, short essay writing, listening comprehension, and dialogue exercises, as shown in Table 2. These assessments help measure students’ language proficiency across different contexts and provide targeted feedback for further learning.

#### 3.1.2. Learning Method Description

Subsequently, the groups were taught and tested according to different teaching methods and various teaching sequences, as shown in Table 3.

As shown in Table 3, three distinct large language models are employed as baseline models: LLama [45], GLM [46], and Bard [47]. These models provide references for evaluating the efficacy of the proposed EEG fusion module and differential LLMs within the automated language learning teaching assessment system. LLama is a large language model developed by Meta (Facebook). Based on the Transformer architecture, LLama processes natural language through multiple layers of self-attention mechanisms. Its structure is similar to OpenAI’s GPT model, using an encoder–decoder framework, which enables the model to perform well in both understanding and generating text. LLama is designed to improve inference efficiency, allowing high performance even with limited computational resources. Due to its flexible architecture, LLama can adapt to various downstream tasks. GLM, or “General Language Model”, was developed by the Knowledge Engineering Group (KEG) at Tsinghua University, aimed at becoming a versatile model suitable for various natural language processing tasks. GLM uses a bidirectional encoder and autoregressive generator architecture. Unlike standard Transformer models, GLM combines bidirectional and autoregressive structures, which enhances its ability to handle complex language tasks involving context. This design enables GLM to generate text while effectively understanding it. Thanks to its combined bidirectional encoding and autoregressive generation architecture, GLM excels in long-text generation and comprehension tasks. Bard, developed by Google, is a dialogue-oriented language model designed to offer a more natural interactive experience. Bard is particularly suited for tasks like dialogue generation, information retrieval, and knowledge-based Q&A. It is built on Google’s Pathways framework, which dynamically adjusts computational resources to handle tasks of varying scales, making it especially effective for dialogues and complex Q&A tasks. Bard uses a large-scale Transformer architecture with deeper layers and a higher parameter count, enhancing its dialogue generation capabilities. Each of these models has strengths across different tasks, and in a language learning evaluation system, their unique features can be combined to enable precise content generation, knowledge supplementation, and personalized adjustments in language comprehension.

The data for the manual teaching group come from traditional teaching methods, with the aim of serving as a control group to compare against the automated teaching based on large language models. This group of students is taught by experienced language teachers who conduct face-to-face lectures and interactive sessions. The content taught is consistent with that of the other groups using language models, covering the same five learning themes (Sports, Travel and Tourism, Restaurants and Food, Shopping and Markets, Health and Medical Care). The core of manual teaching lies in the teacher’s ability to instantly adjust teaching strategies based on student feedback and learning progress, ensuring that students fully understand the material. In practice, teachers use traditional language teaching methods, such as the following: 1. Explanation and Demonstration: The teacher first explains the key vocabulary and sentence structures for each theme, ensuring students understand the meaning and usage of the words. Teachers also demonstrate correct pronunciation and sentence structure, helping students master these through repeated practice. 2. Questioning and Interaction: The teacher interacts with the students through questions, ensuring that every student participates in class discussions. The teacher adjusts the content based on student feedback, focusing on clarifying areas that students find difficult to understand. Through these tests, every effort was made to ensure the fairness and randomness of each teaching method during evaluation.

#### 3.1.3. EEG Data Collection

Regarding the collection and recording of EEG data, this study employed a high-precision EEG headset to ensure comprehensive monitoring of learners’ brain activity. The experiment was conducted in a controlled environment to minimize external interference, enhancing the quality and reliability of EEG data. During the experiment, participants wore electrodes arranged according to the international 10–20 system to ensure accurate recording of activity in various brain regions. EEG data were recorded using real-time data acquisition and synchronized marking. First, the raw signals of brain activity were collected through the electrodes on the headset, with a sampling frequency of 1000 Hz to capture subtle changes in neural activity. To accurately mark learning content and corresponding timestamps, the experimental system was synchronized with the EEG recording device, aligning each learning activity with specific EEG signal timestamps. This synchronization process facilitates subsequent analyses, allowing cognitive states to be correlated with different stages of the learning process.

Finally, the participants in the learning tasks were the same group whose EEG data were collected. All participants wore EEG headsets during the learning process to ensure real-time monitoring and recording of their brain activity during learning activities. This design ensured the synchronization of EEG data with learning activities, enabling each learner’s cognitive state to be accurately matched with the specific learning content they engaged in during the experiment.

#### 3.1.4. EEG Data Preprocessing

**Figure 1:**
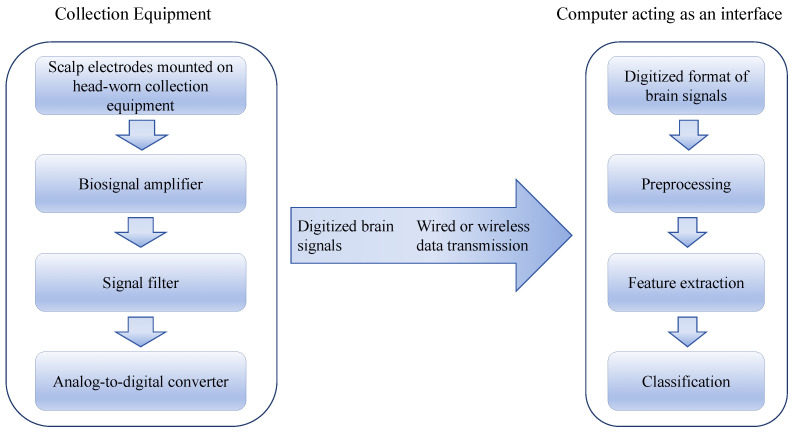
Preprocessing flowchart.

To obtain an EEG dataset for assessing language learning abilities, the experiment meticulously divided the learning process of the participants into two main stages: learning and testing. During the learning phase, participants learned the basics of a new language through large language models. In the testing phase, participants underwent a language proficiency test. The test results were then analyzed in conjunction with the EEG data recorded during the learning process. Each learning phase lasted 10–15 min, with continuous EEG data recording and precise marking of learning content and timestamps. The combination of EEG data from both the learning and testing phases, along with test results, was used to dynamically adjust the large language model’s teaching content and pace to better match each participant’s learning rhythm and abilities. Table 4 shows the technical specifications of the EEG equipment used in the experiment, with a sampling rate (accuracy) of 24-bit:

These EEG data included several key parameters, such as the power spectral density (PSD) within different brainwave frequency ranges, the intensity variations in different brain regions, and the dynamic changes in specific brainwaves (e.g., α waves, β waves, and θ waves). These parameters are closely related to the learner’s attention level, memory encoding ability, and cognitive load, providing critical insights into the neurophysiological activities during the language learning process. During data preprocessing, the raw EEG data were first downsampled from 1000 Hz to 250 Hz, and a 0.5 Hz high-pass and 50Hz low-pass filter were applied to remove low-frequency drift and high-frequency noise. Subsequently, the EEGLAB signal processing tool was used to identify and remove artifact activities (such as eye movement artifacts and electromyographic artifacts), and interpolation was applied to repair bad channels, as shown in Figure 1.

**Figure 2:**
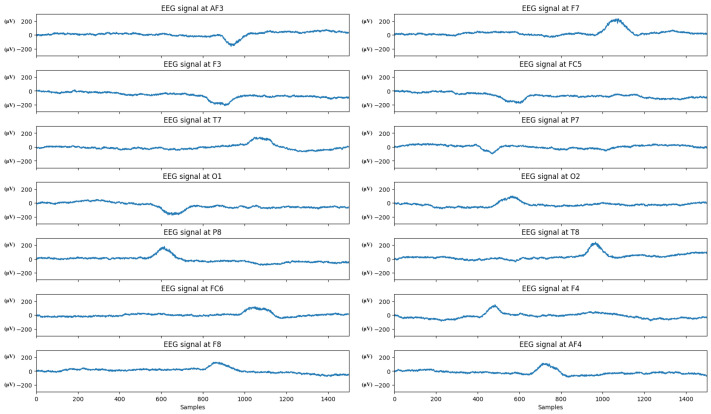
The EEG signals (processed) collected from the test participants in the experiment, with units in μV.

After ensuring data integrity, the EEG data were segmented according to the time periods of the learning and testing phases, with each segment lasting 2 s. This ensured that each participant had at least 30 s of artifact-free valid signals in each phase. The processed data were then synchronized with the timestamps of the learning content, generating a unified dataset available for further analysis, as shown in Figure 2.

### 3.2. Proposed Method

#### 3.2.1. Overall

**Figure 3:**
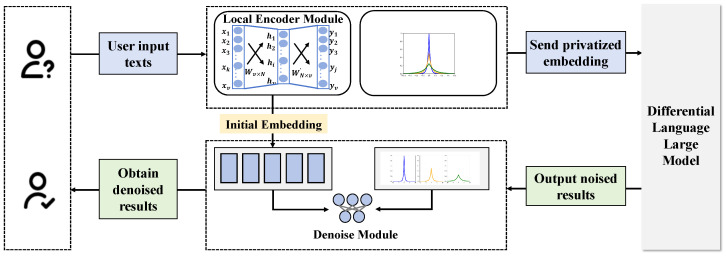
This figure depicts the structure and data flow of the overall model, demonstrating the process from user text input to the differential LLMs, including the local encoding module, denoising module, and the output of results, comprehensively showing the detailed steps of system information processing.

This study proposes an automated language learning teaching assessment system based on EEG and differential LLMs. The system aims to optimize the teaching and assessment processes of language learning by integrating advanced neuroscientific tools and the latest natural language processing technologies. The core idea is to use EEG data to monitor learners’ cognitive states during the language learning process and to apply differential LLMs to personalize learning content, thereby enhancing the specificity and effectiveness of teaching, as shown in Figure 3.

The EEG fusion module is a key component of this system, responsible for collecting and analyzing the brain activity data of learners during the learning process. This module captures the brain’s electrical activity through multiple electrodes mounted on a headgear, monitoring the learner’s cognitive state in real time, including attention focus, information processing, and memory encoding. In implementation, the EEG fusion module first preprocesses the raw EEG signals, including filtering, denoising, and amplification, to improve the quality and usability of the signals. Subsequently, the module employs multidimensional position encoding techniques to encode spatial and temporal information in the EEG data, which helps capture complex patterns in brain activity. After data processing, the EEG fusion module uses machine learning algorithms to extract features from the processed data. These features are then used to train deep learning models to predict learners’ cognitive states and learning effectiveness. Combined with the output from the differential LLM, this module provides real-time feedback on learners’ learning status to teachers, aiding them in adjusting teaching strategies and content.

The differential LLM is another core component that personalizes the adjustment of language learning data based on the specific needs of each learner. Unlike traditional language models, the differential LLM achieves this by designing a unique layer of differential processing. It first analyzes the learning content to identify key knowledge points and challenges for the learner. Then, the model personalizes the teaching content based on the learner’s educational history, cognitive abilities, and personal preferences. For example, for learners with weak language foundations, the model might recommend more basic language exercises and detailed grammar explanations; for advanced learners, it may provide more language application exercises and complex language structure analyses.

The differential loss function is key to optimizing the training process of the differential LLM. Traditional loss functions typically consider only the error between the model’s output and the actual labels, whereas the differential loss function additionally considers the individual differences of each learner, optimizing the model to suit the specific needs of each individual. This loss function focuses not only on the overall educational effectiveness but also strives to reduce the prediction error for individual learners, making the teaching content more aligned with personal learning progress and style. The design of the differential loss function is based on the philosophy that teaching should not be a “one-size-fits-all” approach, but should be flexibly adjusted according to the actual situation of each learner. By continuously analyzing learners’ feedback and progress, combined with the accuracy of model predictions, the differential loss function adjusts model parameters continuously, optimizing teaching strategies to achieve the best educational outcomes.

#### 3.2.2. Differential Language Large Model

**Figure 4:**
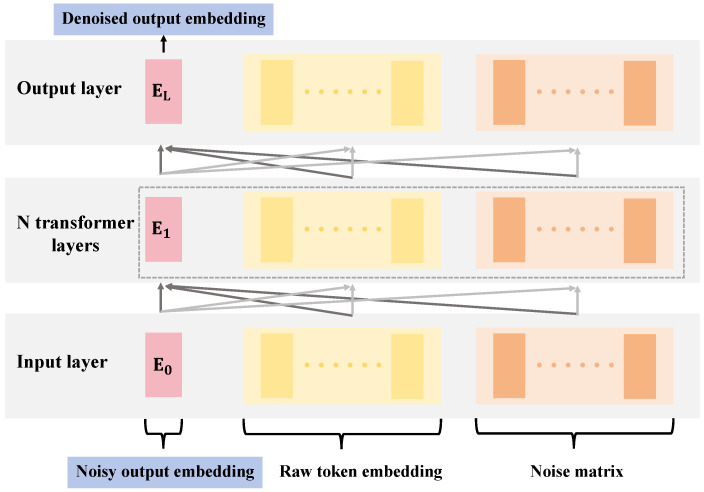
This figure displays the detailed architecture of the differential LLM.

The differential LLM is one of the core technologies in this study, integrating the deep learning Transformer architecture with a specific differential design to meet the personalized learning needs of different learners. The structural design details and parameter settings of this model are key to achieving efficient language learning processing. The differential LLM employs a multi-layer Transformer structure, as shown in Figure 4.

Specifically, the model includes the following main components:

1. Input layer: Responsible for processing raw token embeddings and noise matrices. Token embeddings convert textual data into a format that the model can process, while the noise matrix is key to introducing differential design, enhancing the model’s adaptability to different users’ data by increasing randomness during the training process.
2. Transformer layers: The model includes N Transformer layers, each performing self-attention and feed-forward operations. In this study, a stack of 12 Transformer layers is chosen, with each layer having 12 heads to ensure sufficient model complexity and processing capability.
3. Output layer: The final layer is a linear output layer that converts the output of the last Transformer layer into the final language representation, which will be used for downstream language learning tasks.

The network parameters of this model are set as follows:

- Token embedding dimension: The dimension of each token vector is set to 768, a common setting in current deep learning models, to balance computational complexity and performance.
- Feed-forward network dimension: The width of the feed-forward network within each Transformer layer is set to 3072, ensuring that each layer can learn sufficient features and perform effective non-linear transformations.
- Number of attention heads: The multi-head attention mechanism in each layer is divided into 12 heads, allowing the model to capture different features in multiple subspaces simultaneously, enhancing the model’s learning capabilities.
- Position encoding: A combination of sine and cosine functions is used to add positional information to the input token embeddings, with the dimension of the position encoding matching that of the token embeddings.

This design allows the model to dynamically adjust focus within a given context, effectively extracting and utilizing language information. The design of the differential LLM aims to enhance personalized learning outcomes. Through differential design, the model not only learns universal language rules but also captures specific learning patterns related to individual users, often overlooked in traditional language models. For instance, by analyzing the noise matrix and user feedback, the model can adjust its parameters to better suit specific user needs, thereby providing more precise personalized learning materials and suggestions. Additionally, the multi-layer Transformer structure provides the differential LLM with greater flexibility and accuracy in handling complex language structures. Each layer’s self-attention mechanism can process different parts of the input data in parallel, significantly increasing processing speed and efficiency. The inclusion of feed-forward networks further enhances the model’s capability to handle non-linear relationships, enabling it to effectively undertake not just basic language understanding tasks but also more complex language generation and reasoning tasks.

#### 3.2.3. EEG Fusion Module

**Figure 5:**
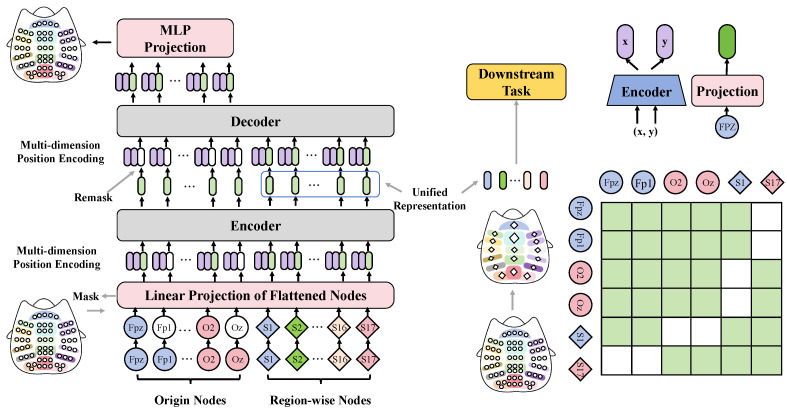
This figure illustrates the detailed architecture of the EEG fusion module, from encoding to decoding the signal, showing how EEG signals are transformed into a unified representation for downstream tasks through multidimensional positional encoding and regional node processing.

The EEG fusion module is one of the core components of this automated language learning teaching assessment system, designed to monitor and analyze learners’ brain electrical activity in real time, providing deep insights into their cognitive states. This module integrates advanced signal processing technologies and machine learning algorithms to optimize teaching strategies and content during the language learning process, as shown in Figure 5. The EEG fusion module consists of the following main parts:

1. Input layer: It first receives raw EEG signal data, which are collected in real time through a multi-channel EEG cap worn on the head. The number of input channels usually corresponds to the number of electrodes on the EEG cap.
2. Preprocessing layer: Raw EEG signals contain a lot of noise and irrelevant information. The purpose of the preprocessing layer is to enhance the signal quality through filtering, artifact removal, and normalization. For example, a bandpass filter may be used to remove signals outside typical brainwave frequencies (such as below 0.5 Hz and above 50 Hz).
3. Feature extraction layer: The signals processed by the preprocessing layer are then fed into the feature extraction layer, which utilizes various algorithms such as Fast Fourier Transform (FFT) and wavelet transform to extract frequency and time domain features. Additionally, statistical methods can be applied to extract other relevant features, such as the mean, variance, and peak values of the signals.
4. Classification and decoding layer: This layer is the core of the module, utilizing deep learning models like Convolutional Neural Networks (CNNs) or Recurrent Neural Networks (RNNs) to parse the extracted features and map them to the learners’ cognitive states. For instance, a CNN with five hidden layers, each containing a different number of convolutional kernels (e.g., 32 in the first layer, 64 in the second), can be designed to progressively extract and compress the feature space.
5. Output layer: Finally, the model’s output is interpreted as the learner’s cognitive state, such as focused attention, active information processing, and memory encoding. This state information is directly used to adjust the teaching content and strategies.

In the EEG fusion module, key mathematical models include signal processing and machine learning algorithms. The preprocessing of the signal can be represented by the following formula:

(6) $$X_{\mathrm{filtered}}=\mathrm{Bandpass}\left(X_{\mathrm{raw}},\;\mathrm{low}=0.5\;\mathrm{Hz},\;\mathrm{high}=50\;\mathrm{Hz}\right).$$

The feature extraction layer might use the FFT formula as follows:

(7) $$X_{\mathrm{fft}}\left(k\right)=\sum_{n=0}^{N-1}x\left(n\right)\cdot e^{-i2\pi kn/N},$$

where x(n) is the nth sample in the time domain, *N* is the total number of samples, and *k* is the index of the frequency domain samples. The design advantage of the EEG fusion module lies in its ability to integrate a variety of signal processing and machine learning technologies to achieve high-precision monitoring and analysis of learners’ brain activity. This highly integrated and automated analysis not only improves the efficiency of data processing but also enhances the accuracy of teaching assessments. In the task of automated language learning teaching assessment, by monitoring learners’ cognitive states in real time, teachers can receive immediate feedback on the learners’ attention and memory states and adjust teaching strategies accordingly. For instance, when learner distraction is detected, the system can automatically prompt the teacher to slow down the teaching pace or modify the content, ensuring that teaching activities are better adapted to the learners’ actual needs.

#### 3.2.4. Loss Function

**Figure 6:**
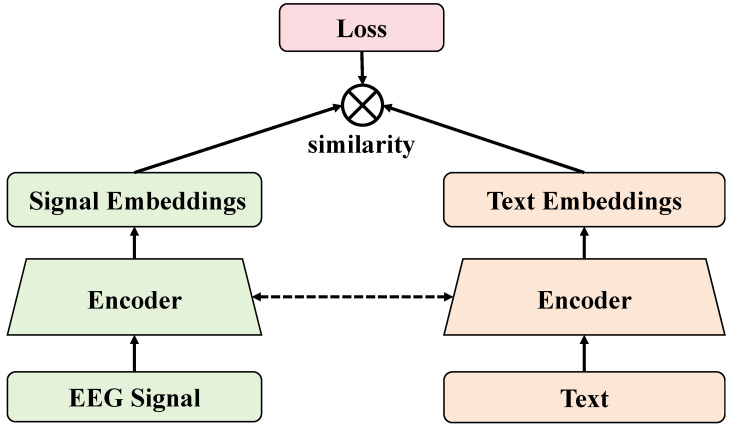
This figure presents the workflow of the loss function module, from the input of specific embedded layers, through the processing by the loss function, to the output of final results, reflecting how the module optimizes differential loss during the learning process to adapt to individual differences.

In traditional Transformer models, the loss function typically used is the cross-entropy loss, which is mainly utilized to calculate the discrepancy between the model’s output and the true labels. This type of loss function is suitable for many classification and prediction tasks and effectively promotes the model to learn the correct classification or prediction of labels. However, this method has limitations in personalized learning applications as it primarily focuses on overall optimization while neglecting to adapt to individual differences, which might not be precise enough when dealing with diverse learner data. The design of the differential loss function aims to address this issue by not only considering the differences between the model output and the true labels but also taking into account the adaptability of the output to individual learner characteristics, as shown in Figure 6.

This design better fits the needs of personalized education, allowing for more precise responses to the unique needs of each learner. The differential loss function combines traditional cross-entropy loss with individual adjustment terms, and its mathematical expression can be represented as

(8) $$L=-\sum_{i=1}^{N}\left[y_{i}\log\left({\hat{y}}_{i}\right)+\lambda \sum_{j=1}^{M}\alpha_{j}\left|f_{j}\left({\hat{y}}_{i}\right)-t_{ij}\right|\right],$$

where *N* is the number of samples, *M* is the number of differential adjustment terms, $y_{i}$ is the true label of the *i*th sample, ${\hat{y}}_{i}$ is the model’s prediction output for the *i*th sample, λ is a hyperparameter that adjusts the weight of the two parts, $\alpha_{j}$ is the weight of the *j*th differential adjustment term, $f_{j}$ is the personalized adjustment function for the model output for the *j*th term, and $t_{ij}$ is the target value for the *i*th sample in the *j*th adjustment item. The differential loss function introduces personalized adjustment items to optimize the specific needs of learners, enabling the model to better adapt to the personalized characteristics of different learners. The key here lies in the adjustment function $f_{j}$ and the target value $t_{ij}$, which respectively represent the method for personalizing the model output and the specific goals of individual learners. Adjustment functions $f_{j}$ are typically designed based on learners’ behaviors or feedback, such as progress in learning or frequency of error types, while the target values $t_{ij}$ are set based on educational objectives and learner characteristics. By optimizing these adjustment items, the differential loss function not only drives the model to learn the correct output but also encourages the model output to align more closely with the actual needs of individual learners.

In the application to this paper’s task—automated language learning teaching assessment—the design of the differential loss function allows the model to consider each learner’s specific performance and needs during the language learning process in greater detail. For instance, for learners with weak language foundations, adjustment items can enhance the learning of basic grammar and vocabulary, while for advanced learners, more complex dialogues and writing exercises can be added. Additionally, the differential loss function also helps improve the relevance and targeting of teaching content. By continuously optimizing the model output, the teaching content becomes more aligned with the actual level and needs of learners, thereby enhancing teaching effectiveness and learner satisfaction.

### 3.3. Experiment Settings

#### 3.3.1. Evaluation Metrics

In the evaluation experiment, the calculation methods for accuracy, precision, recall, and F1-score are as follows:

(9) $$\mathrm{Precision}=\frac{\mathrm{True}\;\mathrm{Positives}}{\mathrm{True}\;\mathrm{Positives}+\mathrm{False}\;\mathrm{Positives}}$$

(10) $$\mathrm{Recall}=\frac{\mathrm{True}\;\mathrm{Positives}}{\mathrm{True}\;\mathrm{Positives}+\mathrm{False}\;\mathrm{Negatives}}$$

This metric reflects whether the model has missed any knowledge points that need to be learned or have not been mastered.

(11) $$\mathrm{Accuracy}=\frac{\mathrm{True}\;\mathrm{Positives}+\mathrm{True}\;\mathrm{Negatives}}{\mathrm{Total}\;\mathrm{Samples}}$$

This metric directly reflects the model’s overall performance across all test data.

(12) $$F1=2\times \frac{\mathrm{Precision}\times \mathrm{Recall}}{\mathrm{Precision}+\mathrm{Recall}}$$

In this experiment, True Positives refer to knowledge points that the model or manual teaching method correctly predicted as mastered and that are indeed mastered. For example, if the system predicts that a learner has mastered a particular vocabulary or sentence structure, and the learner indeed shows mastery in the test, this case is considered a True Positive. For large language model-based teaching, the learner’s mastery is determined by model prediction scores (e.g., correctness in language generation, comprehension scores) and compared against actual test performance. In manual teaching, mastery is determined by the teacher’s assessment and the student’s performance in the test, ensuring that knowledge points are indeed mastered. True Negatives are instances where the system or teaching method predicts a knowledge point as not mastered, and the learner indeed shows no mastery in the test. In large language model teaching, this involves analyzing the model’s predictions of unmastered knowledge points; if the test results also indicate a lack of mastery, it is counted as a True Negative. In manual teaching, unmastered knowledge points are determined by the teacher’s observation and the student’s test performance; if predictions match actual results, they are classified as True Negatives. False Positives are instances where the model or manual teaching method predicts a knowledge point as mastered, but the test reveals that the learner has not mastered it. In large language model teaching, some knowledge points may be predicted as mastered based on model outputs, but a low test score indicates the learner’s lack of mastery. Similarly, in manual teaching teaching, if the teacher judges a knowledge point as mastered but the test score does not meet the standard, it is considered a False Positive. False Negatives are instances where the system or teaching method predicts a knowledge point as not mastered, but the test reveals that the learner has indeed mastered it. In large language model teaching, if the model predicts some knowledge points as unmastered, but test scores indicate correct answers, these are recorded as False Negatives. In manual teaching, if the teacher deems a knowledge point as unmastered, but the student performs well in the test, it is considered a False Negative.

Data Acquisition Methods. Large Language Model Teaching: Each large language model predicts the learner’s mastery of knowledge points based on the learning content. These predictions are compared with subsequent test scores. The test includes vocabulary, grammar, and reading comprehension; scores above the threshold indicate mastery, while scores below the threshold indicate a lack of mastery. The comparison of predictions and test results is used to calculate TPs, TNs, FPs, and FNs. Manual Teaching: In manual teaching, teachers make an initial assessment of the learner’s mastery based on classroom performance, followed by testing. The test includes standardized questions set by the teacher; scores above a certain level are considered mastery, while lower scores indicate a lack of mastery. The comparison between classroom assessments and test results is used to obtain TPs, TNs, FPs, and FNs.

This approach to defining and obtaining True Positives, True Negatives, False Positives, and False Negatives allows for fair and accurate comparisons between the model-based and manual teaching methods.

#### 3.3.2. Test Platform

Regarding hardware configuration, all experiments were conducted on servers equipped with high-performance GPUs (NVIDIA Tesla V100, City of Santa Clara) to ensure computational efficiency. The servers were configured with 128 GB of RAM and 2 TB of SSD storage to support large-scale data processing and model training. Additionally, high-quality EEG data were collected using a BioSemi ActiveTwo v8.14 brain–electrical system, capable of accurately recording brain electrical signals at a high sampling rate of 2048 Hz, ensuring the precision and reliability of the data.

On the software side, all data preprocessing, model training, and testing were conducted in a Python 3.10 environment, primarily relying on the PyTorch 1.8 deep learning framework. Furthermore, EEG data were processed using EEGLAB v2019 and MATLAB 2024a, which facilitated various signal processing and analysis functions, such as filtering, artifact removal, and feature extraction.

In terms of model training strategies, both the differential LLM and the EEG fusion module were trained using supervised learning methods. The differential LLM was trained on labeled language learning tasks comprising tens of thousands of samples, each including textual data and their corresponding language learning performance scores. The models underwent hyperparameter tuning to achieve optimal learning outcomes, with specific settings including a learning rate of 0.001, a batch size of 32, and the use of the Adam optimizer to minimize the cross-entropy loss function. Additionally, early stopping was employed to prevent overfitting, and halting training if there was no improvement in performance on the validation set over ten consecutive training epochs.

To comprehensively assess the performance of the models, *k*-fold cross-validation was utilized, with *k* set to 5. This method maximized the use of limited data resources and provided a more accurate estimation of model performance on unseen data. In each cross-validation run, the dataset was evenly split into five subsets, with each subset sequentially used as the test set while the remaining four subsets served as the training set. This not only enhanced the reliability of model assessments but also helped to understand the model’s performance across different data distributions.

## 4. Results and Discussion

### 4.1. Learning Effect Test Results

The primary objective of this experiment is to evaluate and compare the effects of different teaching methods in language learning, particularly between automated teaching models and traditional manual teaching. The data in Table 5 are calculated from the averages of 200 participants to ensure sample representativeness and data robustness. The specific averaging method is as follows: taking this study’s method as an example, as referenced in Table 3, we sum the test scores from Group A under the “Sports” theme, Group B under the “Travel” theme, Group C under the “Food” theme, Group D under the “Shopping” theme, and Group E under the “Health” theme, and then divide by 200 to obtain the average score.

Initially, traditional manual teaching methods demonstrated high efficacy in the experiment, achieving a precision of 0.91, recall of 0.88, accuracy of 0.89, an F1-score of 0.90, and a test score of 92.75. The manual teaching data are provided for comparison with the performance of our proposed automated language learning and teaching evaluation system, which is based on EEG and differential language models (LLMs). These manual teaching data were collected through a controlled experiment specifically designed for this purpose, aiming to capture the performance of traditional teaching methods under the same experimental conditions. We selected a group of learners with the same number and demographic characteristics as those participating in the automated teaching system test, ensuring consistency in terms of age, learning background, cognitive abilities, and other basic features. This setup allows us to make a direct comparison between the two teaching methods on the same level, thereby fairly evaluating the differences in performance between the automated system and traditional methods. In the controlled experiment, the manual teaching was conducted by experienced language teachers using traditional classroom teaching methods, including face-to-face lectures, paper-based tests, and oral communication. The teachers were instructed to follow a set syllabus, but they could adjust specific teaching strategies and pacing according to the actual teaching situation, simulating real classroom environments. The control group learners received instruction over the same time period as those in the automated teaching experiment, with consistent learning content and assessment methods to ensure data comparability. After the learning sessions, all participants were required to complete the same set of language proficiency tests, designed to assess their abilities across various aspects of language learning, including listening, reading, writing, and speaking.

This outcome reflects the professional capabilities of experienced teachers in language teaching and their flexibility in responding to student feedback, which are often difficult for machine learning models to fully replicate.

For automated teaching models, the LLama model exhibited performance close to manual teaching, with a precision of 0.92 and recall of 0.88, the same accuracy and F1-score as manual teaching, but a slightly lower test score of 91.25. The performance of the LLama model may benefit from its complex deep learning architecture and extensive data training, which allow it to achieve high accuracy and adaptability in language understanding and generation. However, compared to personalized teaching by human teachers, it might still lack in handling some details. The performance of the GLM and Bard models was slightly lower than that of LLama and manual teaching. The GLM model had a precision of 0.85, recall of 0.82, accuracy of 0.83, an F1-score of 0.84, and a test score of 85.75. The Bard model performed slightly better, with a precision of 0.87, recall of 0.84, accuracy of 0.85, an F1-score of 0.86, and a test score of 87.05. These two models might not fully capture the complexity and diversity of language learning due to limitations in model structure or training data. The method proposed in this study, which combines the differential LLM with the EEG fusion module, showed the best results among all methods, achieving a precision of 0.95, recall of 0.92, accuracy of 0.93, an F1-score of 0.94, and a test score of 92.90. This excellent performance is attributable to the personalized learning adjustments of the differential LLM and the real-time cognitive state monitoring of the EEG fusion module, which allow teaching content to be adjusted in real time to match learners’ cognitive and learning needs. This approach enhances the personalization and targeting of teaching, significantly improving learning outcomes.

From the mathematical characteristics of the models, the differential LLM improves the handling of individual differences by incorporating a differential loss function, better adapting to the personalized needs of different learners. The inclusion of the EEG fusion module, by monitoring brainwave activity in real time, precisely adjusts teaching strategies to enhance learning efficiency and effectiveness. This combination of highly personalized teaching methods and advanced technological approaches displays unique advantages in automated language learning teaching assessment. Overall, these experimental results not only validate the effectiveness of various teaching methods but also demonstrate the potential of the differential LLM combined with the EEG fusion module in enhancing language learning outcomes.

### 4.2. Test Result Analysis

**Figure 7:**
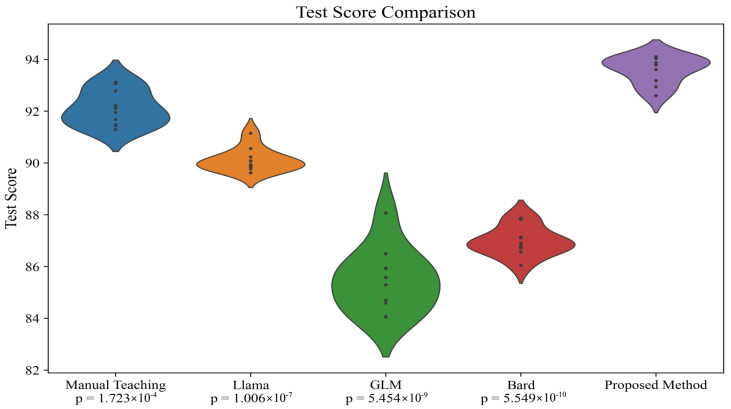
*t*-test on test results in Table 5. Each data point in the figure represents a test item, specifically one of the following: “Vocabulary”, “Sentence Translation”, “Sentence Completion”, “Verb Conjugation”, “Grammar Questions”, “Pronunciation”, “Accent Identification”, “Sentence Construction”, “Listening Comprehension”, or “Basic Conversation”.

**Figure 8:**
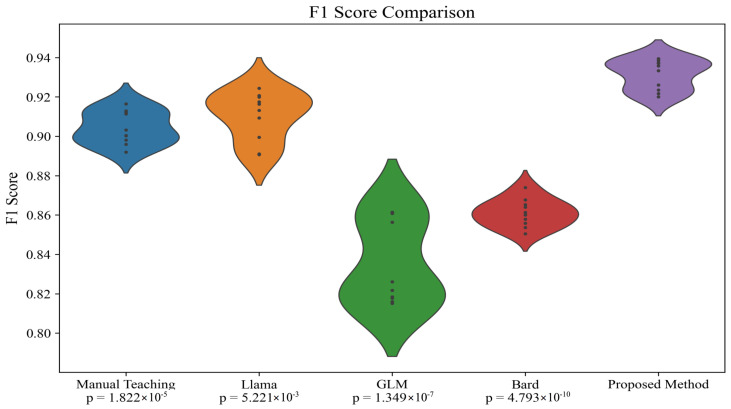
*t*-test on F1-score results in Table 5. Each data point in the figure represents a test item, specifically one of the following: “Vocabulary”, “Sentence Translation”, “Sentence Completion”, “Verb Conjugation”, “Grammar Questions”, “Pronunciation”, “Accent Identification”, “Sentence Construction”, “Listening Comprehension”, or “Basic Conversation”.

In order to investigate the minimal differences in results among the various models presented in Table 5, a more detailed statistical analysis was conducted to further explore this phenomenon. Initially, while the performance differences between the models in terms of precision, recall, accuracy, and F1-score might appear slight, these differences are statistically significant. To verify the statistical significance of these differences, *t*-tests, a common statistical method for comparing the mean differences between two data groups, were implemented. The experiment compared the performance differences between manual teaching methods and our proposed automated language learning teaching assessment system based on EEG and differential LLM, as well as differences within the system models themselves. This approach allowed us to not only test whether the performances of various models were statistically significant but also to verify if our system could achieve the expected enhancement in teaching outcomes when applied in practice. Specifically, we calculated the average performance metrics of each model across different test sets, as shown in Table 2, and conducted paired *t*-tests, as shown in Table 6 and Table 7 and Figure 7 and Figure 8. Each group in Table 6 and Table 7 has only 10 rows of data because the test for all participants included ten items, as shown in Table 2.

The results indicated that although the differences between some models are minor, the method combining differential LLMs with the EEG fusion module demonstrated statistically significant advantages over other models, particularly in terms of precision and F1-score. Moreover, we explored the potential causes for these differences. We believe that the differential LLM can adapt the teaching content based on each learner’s specific learning history and cognitive state, while the EEG fusion module provides real-time feedback on the cognitive state. The combination of these two features allows our system to more precisely adapt to learners’ needs, thus achieving better results in actual teaching applications.

### 4.3. Learning Effect Prediction Results

**Figure 9:**
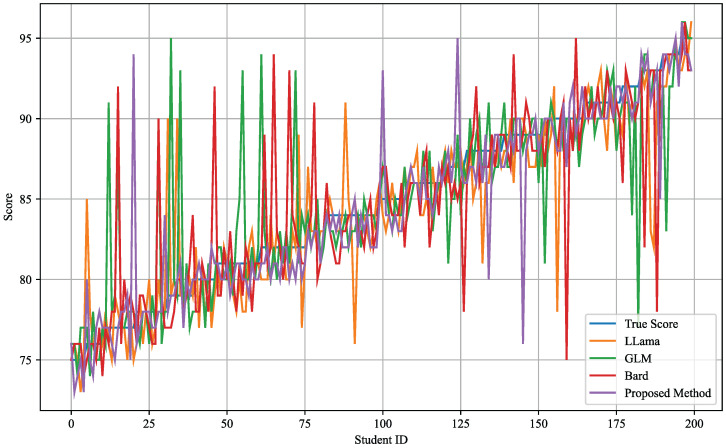
Learning effect prediction curves.

The primary objective of this experiment is to assess and compare the performance of different models in predicting learning outcomes, especially analyzing the fit between these models’ predictions and the actual exam scores. Through quantitative analysis of precision, recall, accuracy, and F1-score, the experiment aims to explore the accuracy and adaptability of different automated teaching models in predicting learning outcomes. The data shown in Table 8 and Figure 9 reflect the performance and advantages of each model in the task of predicting language learning, providing a crucial basis for further optimizing teaching strategies and content.

From the data in the table, it is evident that different models exhibit varied performances in predicting learning outcomes. The LLama model shows high predictive performance, with a precision of 0.93, recall of 0.92, accuracy of 0.94, and an F1-score of 0.92. This performance can likely be attributed to the LLama model’s complex deep learning architecture and extensive data training, which enable it to achieve high adaptability and accuracy in language understanding and generation. The GLM model performs slightly lower, with precision and recall at 0.93 and 0.90, respectively, accuracy at 0.91, and an F1-score of 0.91. Despite the GLM model’s good overall performance, it may not fully capture the complexity and diversity of the learning process in specific language learning tasks due to limitations in model structure or training data. The Bard model performs relatively lower, with a precision of 0.91, recall of 0.88, accuracy of 0.89, and an F1-score of 0.90. This might be related to its specific advantages in generating literary and creative texts, which may limit its ability to precisely predict standardized test scores. The proposed method—integrating the differential LLM with the EEG fusion module—displays the best performance among all models, with a precision of 0.96, recall of 0.95, accuracy of 0.96, and an F1-score of 0.95. This excellent performance is due to the personalized learning adjustments of the differential LLM and the real-time cognitive state monitoring by the EEG fusion module. The differential LLM enhances the model’s adaptability to each learner’s unique learning path through its differential design, thereby improving the accuracy of predictions. The real-time monitoring by the EEG fusion module provides instant feedback on the learners’ cognitive states, allowing for real-time adjustments in teaching content and assessments to match the actual performance and needs of the learners.

Analyzing the mathematical characteristics of the models, the differential LLM can more precisely handle individual learning differences and better predict individual learning outcomes through its differential loss function. At the same time, the EEG fusion module provides deep insights into learners’ cognitive activities through brainwave analysis, which are translated by the model into accurate predictions of learning outcomes. This integration of advanced artificial intelligence technologies with brain science techniques demonstrates unique advantages in automated language learning teaching assessment. It not only enhances the accuracy of teaching and assessments but also boosts the relevance and specificity of the teaching content, thereby significantly improving teaching outcomes and learner satisfaction. Overall, these experimental results not only prove the effectiveness of various teaching models but also reveal the potential of combining the differential LLM and EEG fusion module in enhancing the prediction of learning outcomes. Through this highly personalized teaching strategy, it is possible to more precisely match the specific needs of each learner, thus greatly improving the overall effectiveness of language learning.

### 4.4. EEG Fusion Module Ablation Results

The primary objective of this experiment was to investigate the specific impact of the EEG fusion module on the language learning teaching assessment system through an ablation study. By comparing the performance of models with and without the EEG fusion module, the experiment analyzed the role and effectiveness of this module in different teaching tasks.

As seen in the data from Table 9, models exhibited different results in the presence or absence of the EEG fusion module during test tasks and prediction tasks. In the test task, with the EEG fusion module, the model’s precision was 0.93, recall was 0.90, accuracy was 0.91, F1-score was 0.92, and the score was 90.55. In contrast, without the EEG fusion module, the model performed slightly better with a precision of 0.95, recall of 0.92, accuracy of 0.93, F1-score of 0.94, and a score of 92.90. In the prediction task, with the EEG fusion module, the precision was 0.94, recall was 0.93, accuracy was 0.94, and F1-score was 0.93; without the module, the precision, recall, accuracy, and F1-score were 0.96, 0.95, 0.96, and 0.95, respectively. These data indicate that model performance on various assessment metrics slightly improved upon the removal of the EEG fusion module.

From a theoretical perspective, the EEG fusion module is designed to monitor and analyze learners’ brain activity in real time, providing instant feedback about their cognitive states to the teaching system. Theoretically, this module should enhance teaching content and strategies by accurately monitoring brain activity, thereby increasing the personalization and targeting of instruction. However, the experimental results showed a slight improvement in model performance after the removal of this module. This may be attributed to the fact that although the EEG fusion module can provide in-depth cognitive feedback, its data processing and feature extraction complexity might increase the computational burden on the model, impacting the ultimate teaching effectiveness. Additionally, the noise and uncertainty in EEG data might cause fluctuations in model performance in practical applications.

### 4.5. Limits and Future Works

The automated language learning teaching assessment system proposed in this article, which is based on EEG and differential LLM, has shown significant promise in both theory and practice. However, there are still some limitations that provide directions for future research efforts. First, although the differential LLM combined with the EEG fusion module has demonstrated excellent teaching and predictive performance in experiments, such performance relies heavily on high-quality data inputs and complex algorithmic processing. In practical applications, the complexity and cost of data collection remain significant issues. For instance, the collection of EEG data requires specialized equipment and environments, and participants in experiments must wear electrode caps, which may affect the learners’ experience and comfort. Additionally, EEG data themselves have a high level of noise and require complex preprocessing and analysis to derive effective cognitive state information, demanding considerable computational resources. Therefore, future work needs to focus on simplifying the data collection process, reducing the invasiveness of experimental equipment, and optimizing data processing algorithms to lessen the computational burden. Secondly, although the differential LLM can adjust teaching content based on the characteristics of each learner to achieve personalized learning, its performance largely depends on the quality of model training and the comprehensiveness of the data. Model training currently often requires a large amount of annotated data, which may be difficult to obtain, especially for specific language learning scenarios and particular languages. Finally, the generalizability of the model also needs to be validated across a broader range of language learning tasks and diverse learner populations. Thus, effectively training models with limited or unannotated data to enhance the model’s generalizability and adaptability is a challenge that needs to be addressed in future research.

## 5. Conclusions

This paper aims to explore the application possibilities of an automated language learning teaching assessment system based on EEG and differential LLMs, integrating advanced neuroscience technology and artificial intelligence to offer a new methodology for language teaching and learning effect assessment. This system not only breaks through the limitations of traditional teaching assessment methods but also provides a more precise and personalized teaching support method by real-time monitoring and analysis of the cognitive states of learners.

The innovation of this research is mainly reflected in the following aspects: First, the introduction of differential LLMs provides a new method for the automated teaching system to adjust teaching content based on individual learner differences, which is uncommon in traditional automated teaching models. Secondly, the application of the EEG fusion module enables the teaching system to monitor learners’ brain activities in real time, thereby dynamically adjusting teaching strategies based on learners’ cognitive states, a feature that has also been rare in previous research. Moreover, by combining these two technologies, the system proposed in this paper can provide personalized learning support while offering real-time feedback on learning effects, significantly enhancing the specificity and effectiveness of teaching. Through detailed experimental design, this study has verified the application effects of the EEG fusion module and differential LLM in the automated teaching system. The experimental results show that the method combining differential LLMs and the EEG fusion module exhibits superior performance across various performance indicators. Specifically, in the teaching effect test, this method not only surpasses other automated teaching models in precision, recall, and accuracy but also demonstrates significant advantages in F1-score and test scores. These results adequately demonstrate the potential of integrating EEG technology and differential language models in enhancing teaching quality and learning outcomes.

In conclusion, this research not only provides a new framework for language learning teaching assessment combining EEG technology and artificial intelligence theoretically but also empirically validates the practical application value of this framework. Although there are some limitations and issues that require further exploration in the future, the findings of this research clearly indicate a feasible path to improve teaching methods and enhance teaching effects using the latest technologies, offering significant implications for the future development of the field of language education.

## Figures and Tables

**Table 1:** Grouping method.

	Group A	Group B	Group C	Group D	Group E
Number	40	40	40	40	40
Age	20.8 ± 2.2	21.1 ± 2.4	20.6 ± 2.4	20.9 ± 3.1	21.2 ± 2.7
Gender Ratio (M:F)	22:18	20:20	17:23	19:20	23:17

**Table 2:** Test projects used in this paper.

Test Project	Description
Vocabulary	Identify and spell the learned words.
Sentence Translation	Translate Chinese or English sentences into Spanish.
Sentence Completion	Fill in the correct words or phrases in the sentences.
Verb Conjugation	Correctly conjugate common verbs.
Grammar Questions	Multiple choice or fill-in-the-blank questions.
Pronunciation	Read aloud given words or phrases.
Accent Identification	Identify the placement of accents in words.
Sentence Construction	Use learned vocabulary to construct complete sentences.
Listening Comprehension	Listen to Spanish conversations and answer related questions.
Basic Conversation	Engage in simple conversations.

**Table 3:** Testing procedure for different groups and different teaching methods.

	Sports	Travel	Food	Shopping	Health
Group A	Manual Teaching	LLama	GLM	Bard	Proposed Method
Group B	Proposed Method	Manual Teaching	LLama	GLM	Bard
Group C	Bard	Proposed Method	Manual Teaching	LLama	GLM
Group D	GLM	Bard	Proposed Method	Manual Teaching	LLama
Group E	LLama	GLM	Bard	Proposed Method	Manual Teaching

**Table 4:** EEG Cap parameters.

Technical Indicators	Neuroelectric EEG Cap
Sampling technology	24 bits
Sampling rate	1000
Output format	European Data Format (.edf), raw EEG data, ASCII
Input impedance	l GΩ
LSB resolution	0.05 μV (24-bit)
Observation noise	<1 μV RMS

**Table 5:** Learning Effect Test Results.

Model	Precision	Recall	Accuracy	F1-Score	Test Score
Manual Teaching (control group)	0.91	0.88	0.89	0.90	92.75
LLama	0.92	0.88	0.89	0.91	91.25
GLM	0.85	0.82	0.83	0.84	85.75
Bard	0.87	0.84	0.85	0.86	87.05
Proposed Method	0.95	0.92	0.93	0.94	92.90

**Table 6:** Test result details.

	Test Project	Sports	Travel	Food	Shopping	Health
Group A	Vocabulary	91.00	90.75	84.73	89.13	90.48
	Sentence Translation	92.30	89.25	87.08	86.50	95.18
	Sentence Completion	94.18	88.70	87.48	88.60	95.85
	Verb Conjugation	91.70	90.40	85.48	87.28	95.90
	Grammar Questions	90.73	91.85	83.83	88.40	95.75
	Pronunciation	92.08	89.13	86.15	86.00	93.38
	Accent Identification	93.05	90.85	86.78	85.55	91.50
	Sentence Construction	96.20	88.53	87.73	90.28	96.95
	Listening Comprehension	91.00	87.65	85.68	86.43	94.58
	Basic Conversation	94.55	88.08	83.13	86.45	93.25
Group B	Vocabulary	94.20	92.43	90.10	84.78	86.40
	Sentence Translation	93.10	91.13	90.13	84.40	89.13
	Sentence Completion	93.88	91.50	91.08	85.38	86.33
	Verb Conjugation	93.15	91.18	90.08	83.65	88.00
	Grammar Questions	94.23	90.15	89.68	86.43	88.08
	Pronunciation	92.73	92.80	89.55	84.53	86.10
	Accent Identification	94.28	92.48	90.80	85.40	88.13
	Sentence Construction	93.50	92.55	90.50	85.30	85.75
	Listening Comprehension	92.40	93.95	90.50	88.55	86.13
	Basic Conversation	92.73	91.95	92.38	85.50	85.93
Group C	Vocabulary	85.78	92.05	92.48	90.33	84.83
	Sentence Translation	88.05	94.75	92.15	88.58	85.33
	Sentence Completion	87.15	92.80	90.00	91.08	86.83
	Verb Conjugation	86.08	93.50	91.20	89.25	84.85
	Grammar Questions	87.65	93.10	91.45	90.73	84.45
	Pronunciation	87.18	93.28	91.85	91.30	83.88
	Accent Identification	87.45	94.18	90.40	89.90	84.70
	Sentence Construction	87.05	92.90	93.00	90.68	86.58
	Listening Comprehension	87.53	93.68	94.63	90.30	87.88
	Basic Conversation	86.80	92.53	90.65	92.38	85.23
Group D	Vocabulary	82.70	86.95	94.18	92.35	89.55
	Sentence Translation	85.28	87.80	94.65	91.43	90.18
	Sentence Completion	86.88	86.95	92.68	90.50	90.30
	Verb Conjugation	85.08	87.00	92.50	92.45	90.20
	Grammar Questions	85.05	87.08	93.33	91.98	88.93
	Pronunciation	84.25	86.08	94.00	91.05	91.18
	Accent Identification	86.30	86.88	95.38	91.03	89.85
	Sentence Construction	84.83	86.10	92.80	91.63	90.88
	Listening Comprehension	89.35	85.48	92.50	93.58	90.15
	Basic Conversation	82.83	86.60	92.95	92.13	91.78
Group E	Vocabulary	89.68	83.23	85.30	93.80	92.83
	Sentence Translation	90.70	85.80	87.63	92.80	92.80
	Sentence Completion	91.63	85.90	86.63	94.15	91.03
	Verb Conjugation	88.18	83.90	86.20	93.90	91.90
	Grammar Questions	88.48	86.73	88.18	94.13	92.18
	Pronunciation	89.20	84.70	84.85	92.53	93.60
	Accent Identification	89.78	86.45	85.98	94.80	90.43
	Sentence Construction	88.80	85.30	86.33	92.75	91.95
	Listening Comprehension	90.68	88.93	87.25	94.90	92.45
	Basic Conversation	91.13	83.73	88.10	91.53	91.28

**Table 7:** Test average results.

	Manual Teaching	LLama	GLM	Bard	Proposed Method
Vocabulary	92.218	90.082	84.054	86.712	92.942
Sentence Translation	91.962	89.768	85.578	87.822	94.096
Sentence Completion	91.442	90.558	86.494	87.132	93.872
Verb Conjugation	91.686	89.622	84.592	86.912	93.79
Grammar Questions	91.298	89.934	85.298	87.878	94.108
Pronunciation	92.276	90.072	84.702	86.042	93.184
Accent Identification	91.478	90.236	85.926	86.798	94.028
Sentence Construction	93.066	89.878	85.948	87.102	93.78
Listening Comprehension	93.122	89.856	88.078	86.564	93.612
Basic Conversation	92.112	91.15	84.084	86.776	92.598

**Table 8:** Learning effect prediction results.

Model	Precision	Recall	Accuracy	F1-Score
LLama	0.93	0.92	0.94	0.92
GLM	0.93	0.90	0.91	0.91
Bard	0.91	0.88	0.89	0.90
Proposed Method	0.96	0.95	0.96	0.95

**Table 9:** EEG fusion module ablation results.

Model	Precision	Recall	Accuracy	F1-Score	Score
EEG Fusion Module (Test Task)	0.93	0.90	0.91	0.92	90.55
No EEG Fusion Module	0.95	0.92	0.93	0.94	92.90
EEG Fusion Module (Prediction Task)	0.94	0.93	0.94	0.93	-
No EEG Fusion Module	0.96	0.95	0.96	0.95	-

## Data Availability

The data presented in this study are available on request from the corresponding author.
